# The Protective Effects of a Combination of an Arginine Silicate Complex and Magnesium Biotinate Against UV-Induced Skin Damage in Rats

**DOI:** 10.3389/fphar.2021.657207

**Published:** 2021-06-15

**Authors:** Demet Cicek, Betul Demir, Cemal Orhan, Mehmet Tuzcu, Ibrahim Hanifi Ozercan, Nurhan Sahin, James Komorowski, Sara Perez Ojalvo, Sarah Sylla, Kazim Sahin

**Affiliations:** ^1^ Department of Dermatology, Faculty of Medicine, Firat University, Elazig, Turkey; ^2^ Department of Nutrition, Faculty of Veterinary Medicine, Firat University, Elazig, Turkey; ^3^ Department of Biology, Faculty of Science, Firat University, Elazig, Turkey; ^4^ Department of Pathology, Faculty of Medicine, Firat University, Elazig, Turkey; ^5^ Research and Development, JDS Therapeutics, LLC, Harrison, NY, United States

**Keywords:** UVB, skin, arginine silicate, magnesium, biotin

## Abstract

The purpose of this study was to observe the effects of a novel combination of inositol-stabilized arginine silicate complex (ASI) and magnesium biotinate (MgB) on the prevention of skin damage after UVB exposure in rats. Forty-nine Sprague-Dawley rats were randomized into one of the following groups: (1) NC, normal control, (2) SC, shaved control, (3) UVB (exposed to UVB radiation), (4) ASI+MgB-L (Low Dose), (5) ASI+MgB-H (High Dose), (6) ASI+MgB-L+MgB cream, (7) ASI+MgB-H+MgB cream. The results showed that ASI+MgB treatment alleviated the macroscopic and histopathological damages in the skin of rats caused by UVB exposure. Skin elasticity evaluation showed a similar trend. ASI+MgB increased serum Mg, Fe, Zn, Cu, Si, biotin, and arginine concentrations and skin hydroxyproline and biotinidase levels while decreasing skin elastase activity (*p* < 0.05) and malondialdehyde (MDA) concentration (*p* < 0.001). Moreover, ASI+MgB treatment increased skin levels of biotin-dependent carboxylases (ACC1, ACC2, PC, PCC, MCC) and decreased mammalian target of rapamycin (mTOR) pathways and matrix metalloproteinase protein levels by the regulation of the activator protein 1 (AP-1), and mitogen activated protein kinases (MAPKs) signaling pathways. In addition, ASI+MgB caused lower levels of inflammatory factors, including TNF-α, NFκB, IL-6, IL-8, and COX-2 in the skin samples (*p* < 0.05). The levels of Bax and caspase-3 were increased, while anti-apoptotic protein Bcl-2 was decreased by UVB exposure, which was reversed by ASI+MgB treatment. These results show that treatment with ASI and MgB protects against skin damage by improving skin appearance, elasticity, inflammation, apoptosis, and overall health.

## Introduction

Various intrinsic and extrinsic factors influence skin aging. Internal skin aging, termed ‘spontaneous aging,’ is the physiological changing of the skin affected by genetic factors and occurs naturally over time (Farage et al., 2008). However, skin aging is also associated with exposure to environmental factors, particularly ultraviolet (UV) radiation. Premature aging from prolonged sun exposure is known as ‘photoaging’. Photoaging from chronic UV exposure leads to a complex skin-changing process that occurs predominantly on cutaneous surfaces exposed to the sun (Gragnani et al., 2014). Photoaging mechanisms include free radicals, which lead to the accumulation of reactive oxygen species (ROS). ROS mediates harmful post-translational properties on aging skin by direct chemical alterations to DNA, cell lipids, and dermal matrix proteins, including collagens (Gragnani et al., 2014). All these processes result in an irregular and non-functional accumulation of elastic fibers in the skin. Clinical manifestations of photoaging include wrinkles, telangiectasias, laxity, loss of translucency, and various pigmented spots such as freckles and solar lentigines (Binic et al., 2013; Pandel et al., 2013; Gragnani et al., 2014). Similar to photoaging, chronic exposure to solar radiation causes multiple skin disorders, including sunburn, irregular pigmentation, and skin cancer, specifically non-melanoma skin cancers (Dunaway et al., 2018).

Supplementation with certain nutrients may help prevent and treat skin damage caused by both intrinsic and extrinsic factors. Dietary supplementation with arginine has been shown to facilitate wound healing, enhance insulin sensitivity, collagen deposition, cell proliferation, T-lymphocyte function, protein synthesis, and promote positive nitrogen balance (Dorner et al., 2009; Kalman et al., 2015; Durmus et al., 2017). Silica (Si) is well recognized as effective in preventing skin damage because it plays a significant role in collagen production and connective tissue formation (Seaborn and Nielsen, 2002). Inositol-stabilized arginine silicate complex (ASI) consists of a combination of arginine, silicon, and inositol and has beneficial effects on vascular and bone health (Proctor et al., 2005). The use of arginine in complexes with inositol and silicon increases arginine absorption and silicon (Proctor et al., 2007; Kalman et al., 2015).

Biotin is a vital cofactor for the five biotin-dependent carboxylases, including acetyl-CoA carboxylases 1 and 2 (ACC1 and ACC2), pyruvate carboxylase (PC), 3-methylcrotonyl-CoA carboxylase (MCC), and propionyl-CoA carboxylase (PCC), all of which participate in metabolic processes in mammals (Zempleni et al., 2009; Tong, 2013). The crucial importance of biotin was first revealed when rats fed biotin-free diets developed neuromuscular dysfunction, alopecia, and dermatitis (Wolf, 2005; Mock, 2017). Biotin deficiency leads to various physiological abnormalities, such as disorders of energy metabolism and the immune system and decreased collagen synthesis (Zempleni et al., 2009). Biotin has long been known for its relationship with the skin. Although direct involvement remains unclear, biotin deficiency triggers many symptoms such as seborrheic eczema, hair loss (alopecia), psychosis, opportunistic infections, and various neurological symptoms (Zempleni et al., 2009; Trüeb, 2016; Mock, 2017). Similar to biotin, magnesium is an essential element for an extensive range of cellular progressions such as oxidative phosphorylation, glycolysis, cellular respiration, protein synthesis, and insulin metabolism (Lowney et al., 1995; Yin et al., 2019). Magnesium (Mg) virtually blood vessel smooth muscle cells to block calcium flow (Woods, 1991). Magnesium biotinate (MgB) is a novel biotin salt that is 40 times more soluble than d-Biotin. Preclinical models have shown that MgB is a bioavailable form of biotin that has superior absorption and greater uptake in the tissue compared to d-Biotin (Ojalvo et al., 2019). No previous studies have been conducted to determine the properties and underlying mechanism of a novel combination of ASI and MgB on skin damage in rats chronically exposed to ultraviolet (UV) radiation. Therefore, in the present study, we investigated whether ASI and MgB combination could protect rats from UVB-induced UV damage. The effect of oral and topical applications of ASI and MgB combination on UVB-induced damage was investigated by hematoxylin and eosin, immunohistochemistry, skin elasticity biochemical assay and molecular analyses. The experimental results suggested that the protective mechanism of ASI and MgB combination against UVB-mediated UV damage might act through the MMPs, mTOR , and inflammatory markers, including TNF-α, NFκB, IL-6, IL-8, and COX-2 and apoptotic pathways.

## Material and Methods

### Animals and Experimental Procedures

A total of 49 male Sprague-Dawley rats (age: 8 weeks, mean weight: 180 ± 20 g) purchased from Firat University Experimental Research Center (FUDAM) were used in the study. The rats were maintained in a controlled environment with a 12:12 light-dark cycle, room temperature of 22 ± 2 C and humidity of 55% ± 10, and had *ad libitum* access to feed and water. All experiments were carried out in accordance with the standard ethical guidelines for laboratory animal use and care as defined in the European Economic Community rules (Anonymous, 2010) and with the approval of the Animal Ethics Committee of Firat University (2018/11-126).

The dorsal hair of rats was clipped under anesthesia (2 cm in width x 6 cm in length) prior to the experiment. Forty-nine rats were randomly divided into seven groups with seven rats each, as shown in Supplementary Table S1) NC (normal control; no UVB radiation), 2) SC (shaved control; shaved and no UVB radiation), 3) UVB group (shaved and received UVB radiation), 4) ASI + MgB-L [shaved, received UVB radiation, ASI (140 mg human equivalent dose (HED) = 2.2 mg ASI/rat/d) and low dose MgB (1.5 mg HED of biotin = 24°μgMgB/rat/d], 5) ASI + MgB-H [shaved, received UVB radiation, ASI and high dose MgB (10 mg HED of biotin = 160 μg MgB/rat/d)], 6) ASI + MgB-L + MgB-C [shaved received UVB radiation, ASI, low dose MgB and MgB cream application (2% Mg)], 7) ASI + MgB-H + MgB-C (shaved, received UVB radiation, ASI and high dose MgB and MgB cream application).

The diet used in the study was adapted to comprise spray-dried egg whites as the single protein source. Avidin protein in egg white binds approximately 1.44 mg biotin/kg purified diet, inhibiting biotin absorption (Lewis et al., 2001). All treatments (ASI and MgB) were supplemented daily as an oral supplement, and UVB irradiation was performed five times per week for ten weeks. The normal control shaved control, and UVB rats were orally administered with saline. The oral and topical dosages of both agents were determined as proposed in the literature (Rathman et al., 2003; Kalman et al., 2015; Nourbakhsh et al., 2016).

ASI contains 49.47% arginine, 8.2% silicon, and 25% inositol. Arginine (3.8 g, 21.8 mmol) was added to a vigorously stirred solution of inositol (1.25°g, 6.9 mmol) in potassium silicate [5°ml, 29.8°Be, 8.3% K_2_O (0.52°g, 5.5 mmol), 20.8% SiO_2_ (1.3°g, 21.8 mmol)]. The ASI complex and MgB were provided by JDS Therapeutics, LLC (Harrison, NY, United States ). Both ASI and MgB were dissolved in drinking water and were administered via oral gavage. In the control group, saline was administered by oral gavage after being dissolved in drinking water. Oral gavage and 2% MgB cream were administered 2°h and 30 min prior to the experiment, respectively (Supplementary Table S1).

### UVB Irradiation (Photoaging Modeling)

For the photoaged rat model, rats were exposed to UVB lamps with UVB with emission spectrums between 290 and 320 nm (peak at 310–315 nm) (Waldman UV800, Germany) and UVA emissions in the range of 320–400 nm (peak at 365) four times per week as described in an earlier study (Fan et al., 2015).To determine the minimal erythema dose (MED) of rats in each group, rats were exposed to UVB at doses of 20, 30, 40, 60, 80, and 100 mj/cm^2^ (approximate dosage increase = 40%) on a total area of 24 cm^2^ on the lower back, which comprised a total of six sites measuring 2 cm× 2 cm. MED was accepted as the amount of UV radiation that produced a minimal erythematous reaction with well-demarcated borders 24 h after UVB exposure. Prior to the experiment’s initiation, the average MED of the seven rats in each group was accepted as the baseline MED for each group. Throughout UVB exposure, the rats were kept in the incubator at a 20–30 cm distance from the UVB lamp, and UVB exposure was performed four times a week. The initial radiation dose was 1 MED in the first and second weeks and was increased by 0.2 MED/week over the remaining 10 weeks (range between weeks 3 and 1=1.2–2.6 MED). UV energy was detected with a UV radiometer. The same procedure was performed in the non-irradiated control group; however, the UVB was switched off. ASI and MgB were administered by oral gavage 2 h prior to the study, and topical 2% MgB cream was administered 30 min before the study.

After successful modeling, the rats were anesthetized; their dorsal skin was photographed. At the end of the experiment, all the rats were sacrificed by cervical dislocation, and blood and dorsal skin samples removed from the irradiated area were collected for further analyses. Blood samples were centrifuged at 3000 ×°g for 10°min, and the serum was carefully removed for further analysis. The sera and tissue samples were stored in a deep freeze (Hettich, Germany) at 80°C until analysis. The skin of the back of the animals was observed daily during the irradiation period.

### Evaluation of Dorsal Skin

A blinded observer assessed noticeable variation of rats’ dorsal skin according to assessment criteria shown in Supplementary Table S2 (Kong et al., 2015). Skin elasticity was measured by performing the pinch test according to the adapted protocol described by Tsukahara et al. (Tsukahara et al., 2005). The dorsal skin at the midline of the rat was picked up with fingers as much as possible until the lower limbs were almost off the ground, and then the rat was released from the fingers. Skin elasticity was defined as skin recovery time between the onset and disappearance of the pinch, whereby longer recovery time was accepted to indicate lower skin elasticity (Supplementary Figure S3).

### Biochemical Analysis

Serum glucose, urea-N (BUN), and creatinine concentrations and activities of alanine aminotransferase (ALT) and aspartate aminotransferase (AST) were analyzed with an automated chemistry analyzer (Samsung LABGEO PT10V, Samsung Electronics Co., Suwon, Korea). For hydroxyproline analyses, the dorsal skin tissues were homogenized using 2 ml of 1 N acetic acid, and the homogenates were centrifuged (3.000 × g, 10 min). The hydroxyproline levels were measured in the supernatants using a commercially available assay kit according to the manufacturer’s instructions (Diagnostic Systems Laboratories, TX, United States ). The intra- and inter-assay coefficients of variations (CV) were 4.3 and 6.5%. The tissue was also homogenized and solubilized in 0.1% Triton-X 100, 0.2 M Tris–HCl (pH 8.0) buffer, followed by ultrasonication and by centrifugation (2000 g × 20 min) to obtain supernatants for the elastase enzyme assay. The activity was detected using a commercially available assay kit (Diagnostic Systems Laboratories, TX, United States ) according to the manufacturer’s instructions. The inter-and intra- assay constants were 3.6 and 6.4%. Skin activity of biotinidase was detected using the colorimetric method by measuring p-aminobenzoate liberation from N-biotinyl-p-aminobenzoate as described in detail by Wolf et al. (Wolf et al., 1983). Biotinidase activity is expressed as mU/100 mg protein.

To determine the concentrations of serum levels of Mg, silicon (Si), iron (Fe), copper (Cu), and zinc (Zn) in the sera and tissue samples, 0.3 g of skin and 0.5 ml of serum samples were digested with 5 ml concentrated nitric acid in a Microwave Digestion System (Berghof, Eningen, Germany) for 30 min and diluted 1∶10 with distilled deionized water before analysis. Mineral levels in each sample were measured by flame AAS (AAS, Perkin-Elmer, Analyst 800, Norwalk, CT, United States ) via documented and fully established processes at the 285.2, 248.3, 213.9, 324,8 nm wavelength for Mg, Fe, Zn, and Cu, respectively. The method was confirmed with specialized reference materials, and the precision was 98%. Mineral concentrations in tissues were expressed as wet weights (Sahin et al., 2018).

The serum biotin was analyzed by HPLC (Shimadzu, Kyoto, Japan) as earlier defined, with minor modifications (Lewis et al., 2001; Grotzkyj Giorgi et al., 2012). The reversed-phase column used was a C18-ODS-3 column (250 × 4.6 mm, 5 m), and the biotin-containing chromatography fractions were dried under a stream of nitrogen before the assessment. Serum arginine concentrations were examined by high-performance liquid chromatography (HPLC, Shimadzu, Japan) as defined by Pieper and Dondlinger (Pieper and Dondlinger, 1997). Sera samples were extracted 1:1 in 35% (wt/vol) sulfosalicylic acid dihydrate. After mixing and centrifugation, the supernatant was mixed 1:1 with lithium-D buffer before analysis. The amino acid standard was obtained from Sigma-Aldrich Chemicals (St. Louis, United States ).

For malondialdehyde (MDA) analyses, skin tissues were homogenized in ice-cold phosphate buffer solution for 5 min using both an ultrasonic (Vibra-Cell 130 VCX; Sonics & Materials, Inc., Newtown, CT) and a mechanic (IKA Works, Inc; Wilmington, NC) homogenizers and then centrifuged at 7,000 g for 15 min and the protein content in the supernatant was determined using nanodrop spectrophotometry (MaestroGen, Las Vegas, NV, United States ). Then, an HPLC apparatus of Shimadzu UV–vis SPD-10 AVP detector, a CTO-10 AS VP column, and 30 mM KH2PO4 and methanol (82.5: 17.5, v/v, pH 3.6) at a flow rate of 1.2 ml/min were used (Shimadzu, Japan). Column waste was monitored at 250 nm.

### Molecular Analysis

Dorsal skin protein levels of biotin-dependent carboxylase including ACC1, ACC2, PC, PCC, MCC, and mammalian target of rapamycin 2 (mTOR2; ser2481), p-p70S6K, p4E-BP1, vascular endothelial growth factor (VEGF), sirtuin 1 (SIRT1), matrix metalloproteinase-1 and -3 (MMP-1 and MMP-3), TNFα, NFκB, IL-6, IL-8, COX-2, Bax, Bcl-2 and caspase-3 were measured using the Western blotting technique as defined earlier (Tong et al., 2015). Skin tissues were pooled and homogenized at 4 °C in an extraction buffer and centrifuged at 13,000 × g for 20 min at 4 °C. Protein samples were separated using 10% SDS-Page and transferred onto nitrocellulose membranes for 1 h prior to applying primary antibodies. The following primary antibodies were used: ACC1, ACC2, PC, PCC, MCC, p-mTORC2, p-p70S6K, p4E-BP1, VEGF, SIRT1, MMP-1, MMP-3, TNFα, NFκB, IL-6, IL-8, COX-2, Bax, Bcl-2, caspase-3, AP-1 (p-c-Jun and p-c-Fos), p-p38 MAPK and β-actin (Santa Cruz Biotechnology, CA, United States ). After washing, the membranes were incubated with secondary goat anti-mouse antibodies (Santa Cruz Biotechnology) in Tris-buffered saline containing 0.05% Tween 20 for 1 h, and protein levels were measured densitometrically.

### Histological Examination

Dorsal skin samples (approximately 1 × 0.4 cm in size) were quickly collected at the end of the study and were fixed in a 10% formaldehyde solution, fixed in paraffin, and then cut into sections of 5 µm thickness, as instructed in the literature (Kong et al., 2013). The slides were stained with hematoxylin and eosin (H&E) for routine histological examination and histological assessment of epidermal hyperplasia. The slides were also stained with Masson’s trichrome and Verhoeffelastic stain to evaluate skin elasticity. For epidermal hyperplasia assessment, the epidermal thickness was photographed using an Olympus DP73 camera, and mean epidermal thickness was calculated based on 10 random site measurements on each slide using an optic microscope (Olympus BX53). Fine lines and wrinkles, two of the most important signs of photoaging, were evaluated both macro- and microscopically by examining microfold formation in the tissues stained with hematoxylin and eosin. Solar elastosis is another principal histological sign of photoaging, characterized as dermal collagen breakdown and accumulation of elastotic material, a bluish stain with the appearance of elastin fibers dermal infiltration of inflammatory cells. These changes in collagen were evaluated using Masson’s trichrome stain and Verhoeff elastic stain. The intensity of inflammatory cell infiltration was expressed as the number of cells per mm^2^.

For immunohistochemistry, collected skin tissues were fixed in 10% neutral formalin for 48 h at room temperature. Subsequently, tissue samples were embedded in paraffin and cut into sections (5 μm thick) using a microtome. The sections were then fixed onto slides. The sections were stained using rat-specific MMP-1 (Thermo Fisher Scientific, WA, United States ), COX-2, IL-6, and mTOR (Abcam, Cambridge, MA, United States ) antibodies with the help of an automatic immunohistochemical staining device (Ventana, Tucson, AZ, United States ). We performed quantitative analyses of the immunoreactivities against MMP-1, COX-2, IL-6, and mTOR antibodies using a light microscope (Olympus BX53) equipped with a digital camera (Olympus DP73) and connected to a PC monitor. For MMP-1, COX-2, IL-6, and mTOR, the staining (0, 0%; 1, <25%; 2, 25–50%; 3, 51–75%; and 4, >75%) were scored.

### Statistical Analysis

Statistical analyses were performed using SPSS for Windows version 21.0 (IBM Corp., Armonk, NY, United States ), with a one-way analysis of variance or Kruskal-Wallis test. Then Tukey *post hoc* test or Mann Whitney *U* test were used for multiple comparisons. A *p* value of <0.05 was considered significant.

## Results

### Biochemical Parameters

As shown in Table 1, there were no significant changes in serum glucose, liver (ALT, AST), and kidney (Urea-N, creatinine) function among groups, indicating no hepato-and nephrotoxic effects of the combination of ASI and MgB in rats exposed to UVB (*p* > 0.05). UVB exposure caused a marked decrease in skin hydroxyproline levels and biotinidase activity by 46.1 and 42.8% compared to shaved rats (Table 1; *p* < 0.001). Compared to the UVB group, hydroxyproline and biotinidase levels increased in all ASI + MgB treatment groups (*p* < 0.05). The ASI + MgB-H + MgB-C group showed the greatest increase in hydroxyproline levels (65.8%) and biotinidase activity (68.2%) (*p* < 0.05) (Table 1). Elastase activity in the dorsal skin increased from UVB irradiation by 53.3% compared to shaved rats (*p* < 0.05), whereas ASI + MgB treatments exerted protection against the increase in dorsal skin elastase activity induced by UVB irradiation (*p* < 0.05). The effect was more pronounced in the ASI + MgB-H group compared to the ASI + MgB-L group. However, a combination of ASI + MgB oral administration and MgB cream provided even greater preventive effects, resulting in a 30.6% decrease in elastase levels compared to the UVB group. We also examined the effects of the combination of ASI and MgB on the MDA levels, a lipid peroxidation marker, induced by UVB radiation in the skin tissue of rats. The concentration of MDA was higher in the UVB-treated group than in the no radiation groups, whereas the concentration decreased significantly in the skin of ASI and MgB-treated groups (*p* < 0.001). The effect of the high dose of MgB combined with ASI and MgB cream was much better compared to the low doses (Table 1).

**TABLE 1:** Effect of inositol-stabilized arginine silicate complex (ASI) and magnesium biotinate (MgB) on serum biochemical parameters, skin hydroxyproline, elastase, biotinidase and MDA levels in rats with UVB-induced photoaging.

Items	Groups						
	NC	SC	UVB	ASI + MgB-L	ASI + MgB-H	ASI + MgB-l + MgB-C	ASI + MgB-H + MgB-C
Glucose, mg/dL	92.57 ± 2.50	91.00 ± 3.38	93.86 ± 2.96	94.00 ± 2.18	90.57 ± 3.26	92.14 ± 1.72	91.43 ± 1.72
BUN, mg/dL	20.60 ± 0.85	20.96 ± 0.47	20.46 ± 1.40	20.41 ± 1.01	21.20 ± 0.94	20.07 ± 1.04	20.59 ± 0.94
Creatinine, mg/dL	0.11 ± 0.01	0.12 ± 0.01	0.13 ± 0.01	0.11 ± 0.02	0.11 ± 0.02	0.12 ± 0.01	0.12 ± 0.01
ALT, U/L	81.29 ± 1.48	82.43 ± 2.28	93.71 ± 3.20	87.00 ± 2.65	84.29 ± 2.83	86.29 ± 3.01	83.43 ± 3.13
AST, U/L	116.86 ± 5.19	118.29 ± 3.27	124.86 ± 5.10	121.14 ± 2.94	120.43 ± 3.43	121.57 ± 3.31	120.29 ± 5.02
Hydroxyproline g/100 g tissue	5.89 ± 0.03^a^	5.86 ± 0.02^a^	3.16 ± 0.03^e^	3.78 ± 0.04^days^	4.51 ± 0.04^c^	4.59 ± 0.06^c^	5.24 ± 0.06^b^
Elastase pmol nitroaniline/hr	4.19 ± 0.04^g^	4.50 ± 0.05^f^	6.90 ± 0.03^a^	6.28 ± 0.04^b^	5.87 ± 0.02^c^	5.70 ± 0.03^d^	4.79 ± 0.04^e^
Biotinidase,mU/100 mg protein	5.83 ± 0.04^a^	5.56 ± 0.06^b^	3.18 ± 0.07^f^	3.57 ± 0.03^e^	4.31 ± 0.11^d^	4.93 ± 0.04^c^	5.35 ± 0.03^b^
MDA, nmol/mg protein	2.59 ± 0.18^f^	2.78 ± 0.14^ef^	7.75 ± 0.29^a^	5.74 ± 0.20^b^	4.61 ± 0.28^c^	4.34 ± 0.16^cd^	3.52 ± 0.11^de^

ALT, alanine aminotransferase; ASI, inositol-stabilized arginine silicate complex; AST, aspartate aminotransferase; BUN, blood urea nitrogen; MgB-C, magnesium biotinate cream; MgB-H, magnesium biotinate high dose; MgB-L, magnesium biotinate low dose; NC, normal control; TP, total protein; SC, shaved control; UVB, ultraviolet B-induced photoaging. Data presented as the mean and standard error. ^a,b,c,d,e,f,g^ Mean values within a row with unlike superscript letters were significantly different (*p* < 0.05).

### Magnesium, Iron, Zinc, Copper, Silicon Biotin and Arginine Analyses

Serum Mg, Fe, Zn, Cu, and Si levels were significantly reduced by 24.9, 15.4, 19.0, 29.3, and 30.2% in the UVB group compared with the shaved group, respectively (*p* < 0.001; Table 2). ASI + MgB groups presented a significant increase in these parameters compared to the UVB group, particularly in the ASI + MgB-H + MgB-C group (*p* < 0.05). Mg, Fe, Zn, Cu, and Si levels increased 165.7, 15.8, 9.0, 23.9, and 237.0%, respectively, compared to the UVB group. In addition, serum biotin and arginine concentrations were decreased by 36.8 and 23.5% in the dorsal skin following UVB irradiation (*p* < 0.01). However, the combination of ASI and MgB treatment improved this reduction caused by UVB (*p* < 0.05; Table 2). This effect was greater in the ASI + MgB-H + MgB-C group (2.2 mg ASI + 160 μg MgB), with biotin and arginine levels increased by 179.5 and 89.8%, respectively.

**TABLE 2:** Effect of inositol-stabilized arginine silicate complex (ASI) and magnesium biotinate (MgB) on serum minerals, biotin, and arginine levels in rats with UVB-induced photoaging.

Item	Groups						
	NC	SC	UVB	ASI + MgB-L	ASI + MgB-H	ASI + MgB-L + MgB-C	ASI + MgB-H + MgB-C
Mg, mg/dL	2.77 ± 0.04^c^	2.33 ± 0.12^d^	1.75 ± 0.06^e^	3.64 ± 0.15^b^	4.84 ± 0.08^a^	3.68 ± 0.13^b^	4.65 ± 0.08^a^
Fe, µg/dL	499.00 ± 25.39^a^	469.00 ± 19.35^ab^	396.86 ± 19.84^b^	436.71 ± 15.53^ab^	457.29 ± 18.36^ab^	442.57 ± 15.42^ab^	459.71 ± 23.88^ab^
Zn, µg/dL	185.71 ± 3.74^a^	187.29 ± 5.71^a^	151.71 ± 4.83^b^	158.29 ± 2.97^b^	166.00 ± 2.63^b^	156.57 ± 2.62^b^	165.43 ± 2.53^b^
Cu, µg/dl	246.71 ± 6.67^a^	254.86 ± 6.88^ab^	180.14 ± 4.12^e^	208.43 ± 6.91^cd^	225.00 ± 5.13^bc^	211.71 ± 4.73^cd^	223.29 ± 5.05^bc^
Si, µg/L	229.49 ± 3.89^b^	227.43 ± 9.23^b^	158.71 ± 3.19^c^	529.43 ± 5.49^a^	532.29 ± 3.52^a^	532.71 ± 5.64^a^	534.86 ± 8.51^a^
Biotin, nmol/L	89.32 ± 2.41^c^	88.70 ± 1.86^c^	56.46 ± 3.59^d^	118.84 ± 3.04^b^	156.94 ± 3.00^a^	119.62 ± 2.90^b^	157.78 ± 4.71^a^
Arginine, nmol/L	1.79 ± 0.01^b^	1.74 ± 0.02^b^	1.37 ± 0.02^c^	2.58 ± 0.04^a^	2.57 ± 0.04^a^	2.54 ± 0.04^a^	2.60 ± 0.04^a^

ASI: inositol-stabilized arginine silicate complex; MgB-C: magnesium biotinate cream; MgB-H: magnesium biotinate high dose; MgB-L: magnesium biotinate low dose; NC: normal Control; SC: shaved Control; UVB: ultraviolet B-induced photoaging. Data presented as mean and standard error. ^a,b,c,d,e^ Mean values within a row with unlike superscript letters were significantly different (*p* < 0.05).

### Macroscopic Appearance

Illustrative pictures of dorsal skin lesions caused by UVB exposure are shown in Supplementary Figures S1, S2. The intensity of fine lines and wrinkles, which is one of the most critical signs of photoaging, was increased by UVB irradiation, with the highest intensity observed in the UVB group (Supplementary Figures S1, S2). In addition, the dorsal skin of the rats in the UVB groups had an irregular and corrugated appearance compared to the control group. In contrast, non-irradiated rats in the normal and shaved groups showed neither wrinkles nor lesions. This not only further demonstrated UVB’s skin-damaging effects but also showed that shaving had no macroscopic damage to the skin. Compared to the UVB group, the skin of rats in the ASI + MgB-H + MgB-C, ASI + MgB-L + C, and ASI + MgB-H groups appeared much smoother without visible lesions. In contrast, the dorsal skin of rats in the ASI + MgB-L group showed numerous wrinkles and mild erythema. At the end of the experiment, rats exposed to UVB with no other treatment had greater visual scores, demonstrating greater impairment when compared to control rats (*p <* 0.001; Supplementary Figure S1). However, the visual scores of the skin of the rats were significantly reduced by 37.8–76.0% in the ASI + MgB groups, an effect that was greatest in the ASI + MgB-H + MgB-C group (−76.0%) (*p* < 0.05; Supplementary Figure S1B).

### Skin Elasticity

The pinch test evaluated rats’ dorsal skin elasticity, and the representative pictures of skin after being pushed are presented in Supplementary Figure S3A. The pinch test indicated no difference between the rats’ recovery times in the normal and shaved control groups (*p* > 0.05). However, skin recovery time, associated with photoaging and a loss of skin elasticity, increased in all UVB groups. Skin recovery time was longest in the UVB group, with a time 6.8 times longer than that of the control group (*p* < 0.05) (Supplementary Figure S3B). Compared with the UVB group, treatment with ASI + MgB significantly reduced recovery time by 34.8–62.8%, with the lowest duration found in the ASI + MgB-H + MgB-C group (−62.8%).

### Histological Examination

**FIGURE 1:**
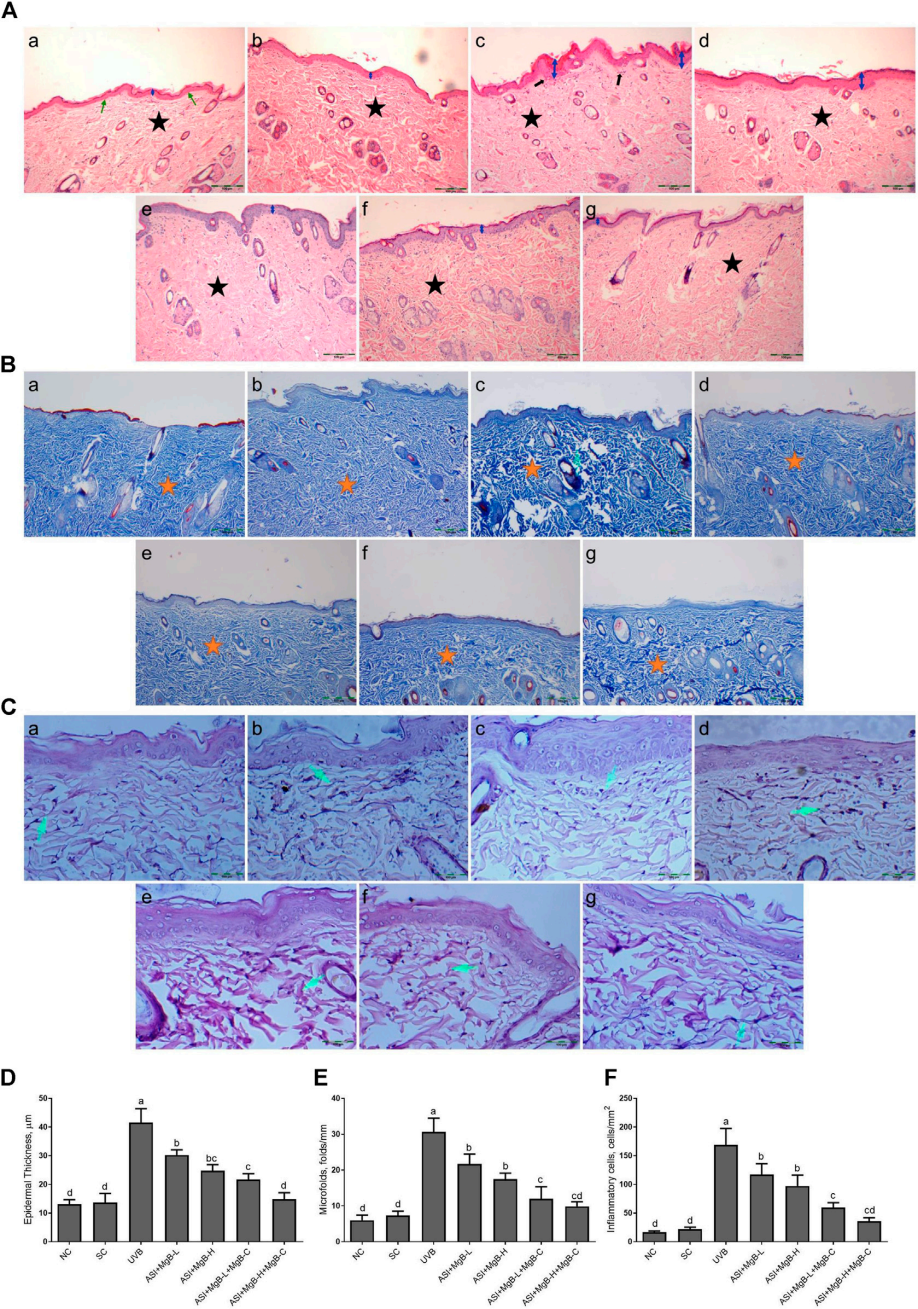
Effect of inositol-stabilized arginine silicate complex (ASI) and magnesium biotinate (MgB) on histopathological changes of skin tissue stained with hematoxylin and eosin (H&E, 100X; **A**), showing relative skin thickness, Masson’s trichrome (MT, 100X; **B**), and Elastin (E, 400X; **C**), and epidermal thickness **(D)**, microfolds **(E)**, and inflammatory cells **(F)** in rats with UVB-induced photoaging. In histopathological images, the groups are shown as **a:** NC (normal control; normal histology of epidermis and dermis (arrow) and tightly arranged collagen fibers (star)); **b:** SC [shaved control; normal histology of epidermis and dermis (arrow) and tightly arranged collagen fibers (star)]; **c:** UVB (An increase in epidermis thickness (double arrow) and loosely arranged dermal collagen fibers (star); **d**: ASI + MgB-L; **e:** ASI + MgB-H; **f:** ASI + MgB-L + MgB-C; **g:** ASI + MgB-H + MgB-C; (ASI and MgB treatments alleviated UVB-induced skin damage features, particularly ASI + MgB-H + MgB-C, showing close to normal epidermal morphology (arrow) and close to normal arranged collagen fibers (star). Masson’s trichrome staining shows collagen (arrow and stars). Elastin staining **(C)** shows decreased collagen fibers (arrows) in UVB, and treatments alleviated the damages. Each bar represents the mean and standard error of the mean. **a–d**: Values within the bars with different superscripts are significantly different (One-way ANOVA and Tukey's *post-hoc* test, *p* < 0.05).

As established in H&E staining pictures (Figure 1A), Masson’s trichrome (Figure 1B), and elastic fiber staining images (Figure 1C), normal and shaved rats presented almost similar appearances and showed normal skin layers, including normal epidermises covered by thin layer stratum corneum, wavy dermal-epidermal junction lines, regularly disseminated hair follicles, closely refined sequential collagen bundles, as well as well-distributed elastic fibers. Besides, no inflammatory infiltration was detected in these groups. After UVB-irradiation, rats’ epidermises exhibited irregular hyperplasia with thickened stratum corneum. Thin and irregular collagen fibers were also observed in the dermis, showing prominent fractures (Figure 1A). In some instances, the elastic fibers were twisted, broken, or even irregularly accumulated, and the density was significantly reduced (Figure 1C).

Moreover, inflammatory infiltrations were visibly demonstrated throughout the dermis. However, ASI + MgB treatment orally or MgB topically (especially at a high dose with cream application) significantly alleviated these UV-induced skin damage features. The rats' skin in ASI + MgB-H + MgB-C revealed fairly complete epidermises and dermis, containing thin layer stratum corneum and well-regulated collagen and elastic fibers uniform thickness and distribution.

In Masson’s trichrome staining, collagen is indicated as the blue-stained parts of the dermis that are not exposed to UVB. The collagen dispersal in the control group was regularly concentrated in the dermis, but the collagen dispersal was changed and destructed with UVB exposure. However, the administration of ASI + MgB prevented collagen fibers from UVB damage. Particularly, the high dose of ASI + MgB with the application of MgB cream presented stronger blue staining as compared to the control group (Figure 1B).

Epidermal thicknesses and microfolds of rats in the UVB group dramatically increased after chronic UVB exposure, with levels 3.0 and 4.2 times that of shaved rats, respectively (Figure 1). However, the epidermal thickening was markedly decreased to 27.2, 40.3, 47.7, and 64.3% in the ASI + MgB-L, ASI + MgB-H, ASI + MgB-L + C, and ASI + MgB-H + C groups, respectively, compared to the UVB group (Figure 1D; *p* < 0.05). Microfolds decreased by 29.3, 42.9, 60.9, and 67.9% in the ASI + MgB-L, ASI + MgB-H, ASI + MgB-L + C and ASI + MgB-H + C groups, respectively, compared to the UVB group (Figure 1E; *p* < 0.05). The intensity of inflammatory cell infiltration in the skin was 7.7 times greater in the UVB irradiation group compared to shaved controls (Figure 1F; *p* < 0.001). The administration of a combination of ASI and MgB treatment partially prevented inflammatory cell infiltration (*p* > 0.05), particularly in the ASI + MgB-H + C group, where infiltration decreased by 79.0%.

**FIGURE 2:**
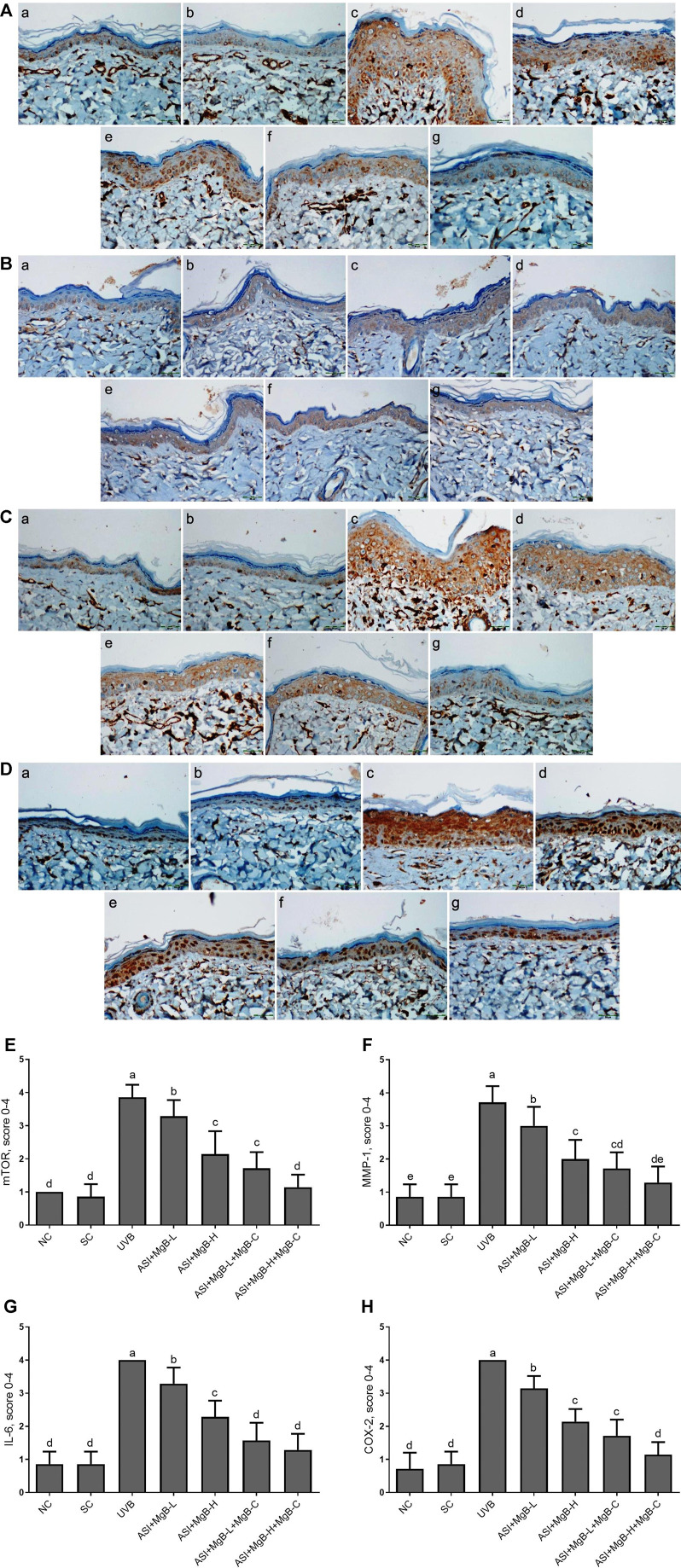
Effect of inositol-stabilized arginine silicate complex (ASI) and magnesium biotinate (MgB changes of skin tissue stained with immunohistochemical stain for the mammalian target of rapamycin (p-mTOR; **A**), matrix metalloproteinase-1 (MMP-1; **B**), interleukin 6 (IL-6; **C**), and cyclooxygenase (COX-2; **D**) in rats with UVB-induced photoaging. For mTOR **(E)**, MMP-1**(F)**, IL-6 **(G)**, and COX-2**(H)**, the staining (0, 0%; 1, <25%; 2, 25–50%; 3, 51–75%; and 4, >75%) were scored. In immunohistochemical images, the groups are shown as **a:** NC, normal control; **b:** SC, shaved control; **c:** UVB; **d**: ASI + MgB-L; **e:** ASI + MgB-H; **f:** ASI + MgB-L + MgB-C; **g:** ASI + MgB-H + MgB-C. **a–e**: Values within the bars with different superscripts are significantly different (Kruskal-Wallis and Mann Whitney *U* test, *p* < 0.05).

The effect of MgB and ASI complex treatment on the expressions of mTOR, MMP-1, IL-6, and COX-2 proteins in the skin of rats was evaluated using immunohistochemistry after UVB exposure (Figure 2). The results showed that mTOR, MMP-1, IL-6, and COX-2 immunoreactivity was increased in the UVB treatment rats skin tissues compared to the control and shaved groups (Figures 2E–H; *p* < 0.001), whereas ASI + MgB-H treatments decreased the expression of mTOR, MMP-1, IL-6, and COX-2 in UVB treatment rats skin tissues. The highest level of decrease was detected in a high dose of MgB combined with ASI and MgB cream group.

### Protein Levels

**FIGURE 3:**
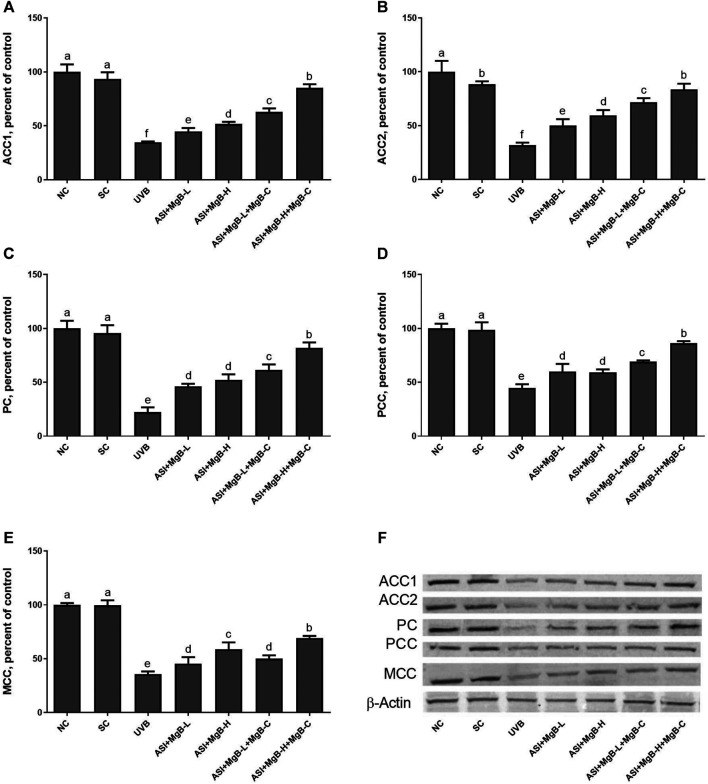
Effect of inositol-stabilized arginine silicate complex (ASI) and magnesium biotinate (MgB) on acetyl CoA carboxylase 1 (ACC-1; **A**), acetyl CoA carboxylase 2 (ACC-2; **B**), pyruvate carboxylase (PC; **C**), propionyl-CoA carboxylase (PCC; **D**), 3-methylcrotonyl-CoA carboxylase (MCC; **E**) protein levels in rats with UVB-induced photoaging. Data are expressed as a percent of the control value. Each bar represents the mean and standard error of the mean. Blots were repeated at least 3 times. Western blot analysis was performed with actin included ensuring equal protein loading **(F)**. **a–f**: Values within the bars with different superscripts are significantly different (One-way ANOVA and Tukey;s *post-hoc* test, *p* < 0.05).

**FIGURE 4:**
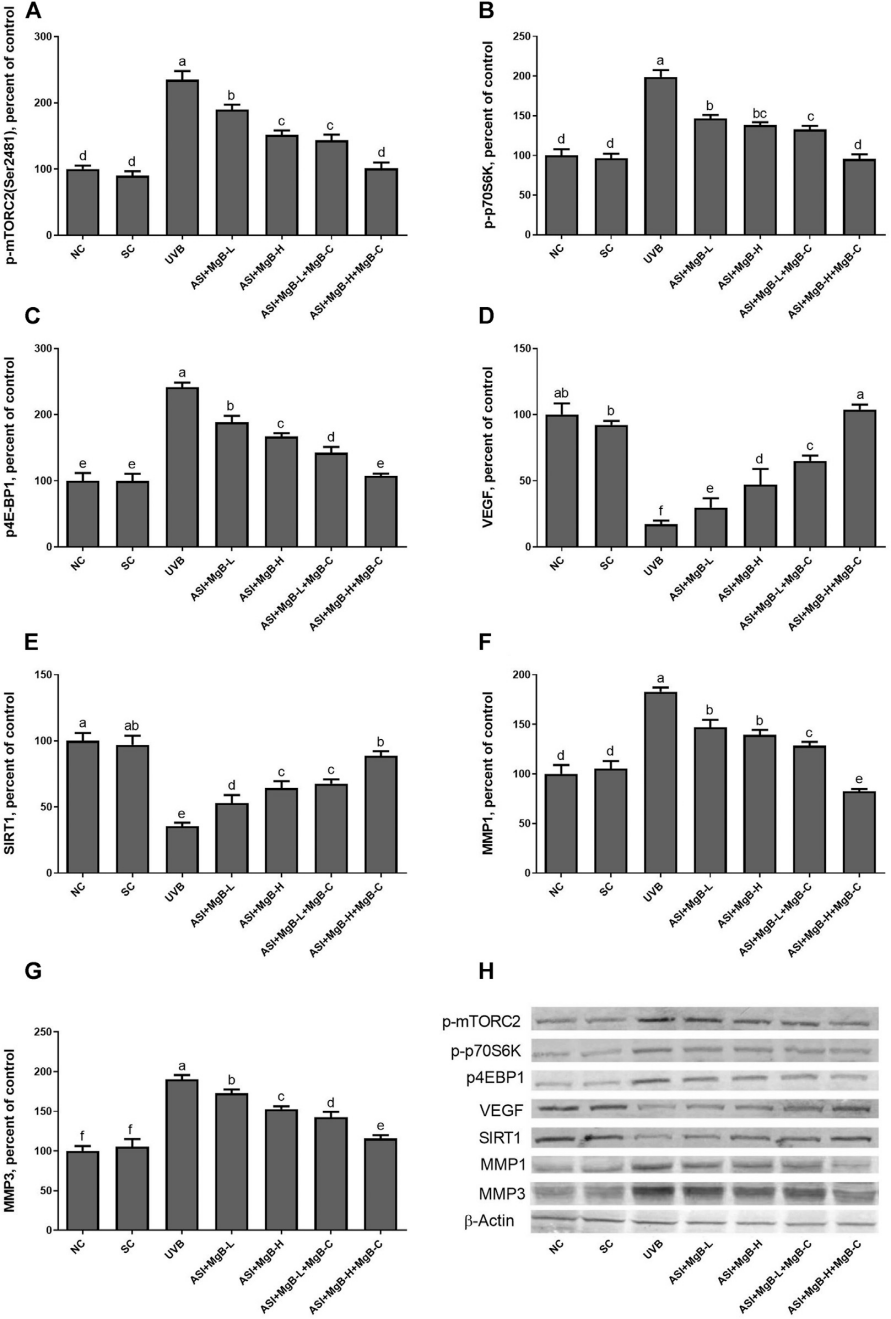
Effect of inositol-stabilized arginine silicate complex (ASI) and magnesium biotinate (MgB) on the mammalian target of rapamycin comple×2 (p-mTORC2; **A**), ribosomal protein S6 kinase beta-1 (p-p70S6K; **B**), eukaryotic initiation factor 4E-binding protein 1 (p4E-BP1; **C**), vascular endothelial growth factor (VEGF; **D**), sirtuin 1 (SIRT1; **E**), matrix metalloproteinase-1 (MMP-1; **F**), and matrix metalloproteinase-3 (MMP-3; **G**) protein levels in rats with UVB-induced photoaging. Data are expressed as a percent of the control value. Each bar represents the mean and standard error of the mean. Blots were repeated at least 3 times. Western blot analysis was performed with actin included ensuring equal protein loading **(H)**. **a–f**: Values within the bars with different superscripts are significantly different (One-way ANOVA and Tukey’s *post-hoc* test, *p* < 0.05).

**FIGURE 5:**
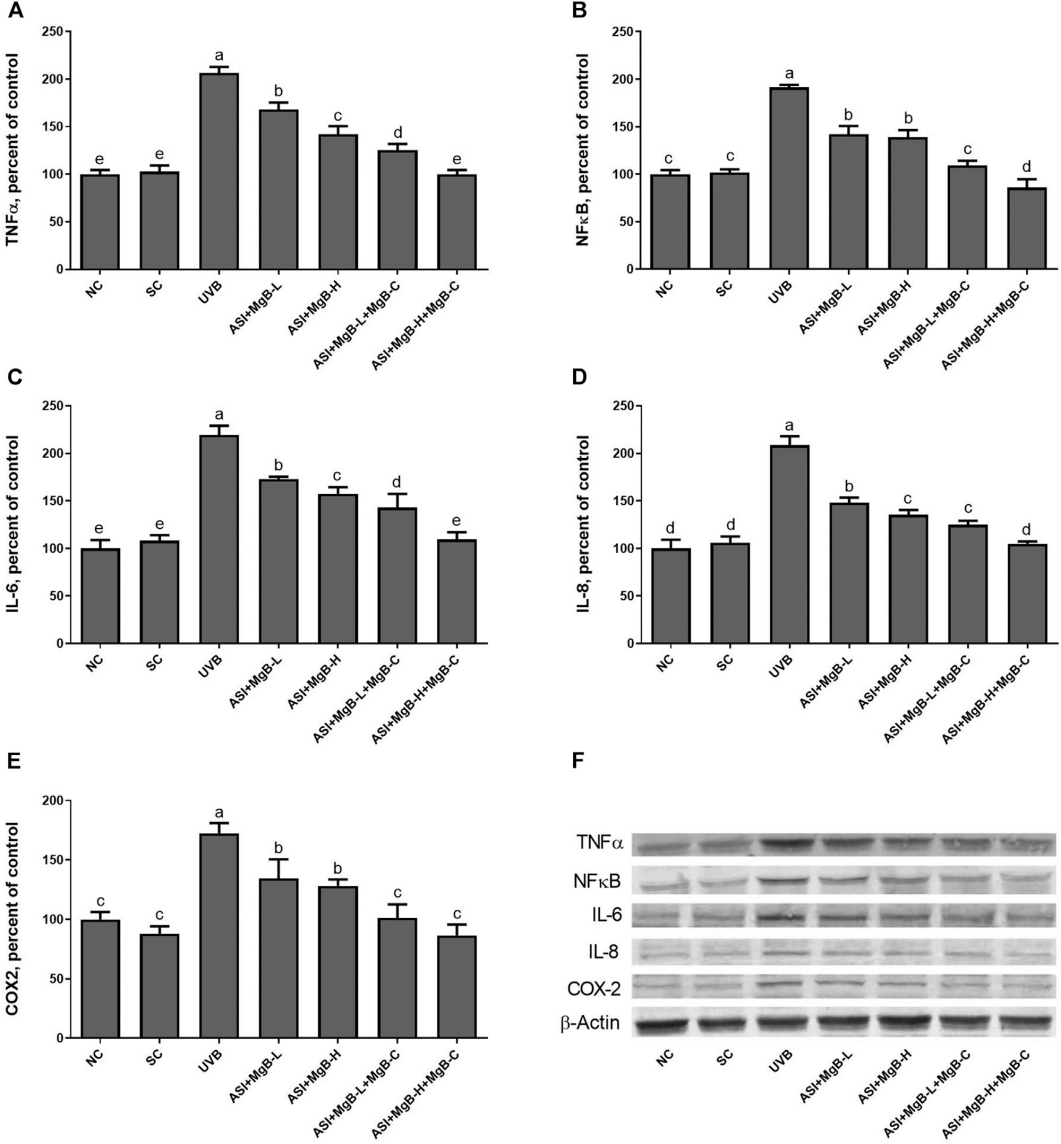
Effect of inositol-stabilized arginine silicate complex (ASI) and magnesium biotinate (MgB) on tumor necrosis factor-alpha (TNF-α; **A**), nuclear factor-kappa B (NF-κB; **B**), interleukin 6 (IL-6; **C**), interleukin 8 (IL-8; **D**), and cyclooxygenase (COX-2; **E**) protein levels in rats with UVB-induced photoaging. Data are expressed as a percent of the control value. Each bar represents the mean and standard error of the mean. Blots were repeated at least 3 times. Western blot analysis was performed with actin included ensuring equal protein loading **(F)**. The data are percentages of the control. **a–e**: Values within the bars with different superscripts are significantly different (One-way ANOVA and Tukey’s *post-hoc* test, *p* < 0.05).

Compared to shaved rats, dorsal skin ACC1, ACC2, PC, PCC, and MCC protein levels decreased by 63.0, 64.0, 76.8, 54.6, and 64.2, respectively, due to UVB irradiation (Figure 3: *p* < 0.001 for all). However, a combination of ASI and MgB treatment, especially the ASI + MgB-H with cream treatment, increased ACC1, ACC2, PC, PCC, and MCC relative to those in the UVB group (*p* < 0.05 for all). To investigate the mechanism underlying the protective effect of ASI + MgB on UVB-induced photoaging, dorsal skin levels of VEGF, SIRT1, mTORC2 (ser2481), p-p70S6K, p4E-BP1, MMP-1, and MMP-3 were also analyzed by Western blot (Figure 4). The results showed that UV irradiation-induced a decrease in protein levels of VEGF and SIRT1 by 81.4 and 63.4% and an increase in p-mTORC2 (ser2481), p-p70S6K, p4E-BP1, MMP-1, and MMP-3 protein levels by 160.4, 106.6, 141.6, 73.7 and 80.9% as compared to shaved rats, respectively. However, ASI + MgB treatment caused a significant increase in protein levels of VEGF and SIRT1 and a decrease in mTORC2 (ser2481), p-p70S6K, p4E-BP1, MMP-1, and MMP-3 protein levels in the dorsal skin of rats exposed to UVB. In addition, the UVB irradiation caused higher levels of inflammatory factors, including TNF-α, NFκB, IL-6, IL-8, and COX-2 protein levels in rats’ dorsal skin (*p* < 0.01; Figure 5). However, ASI + MgB treatment, especially in the high dose with MgB cream application, markedly inhibited their levels (*p* < 0.05).

**FIGURE 6:**
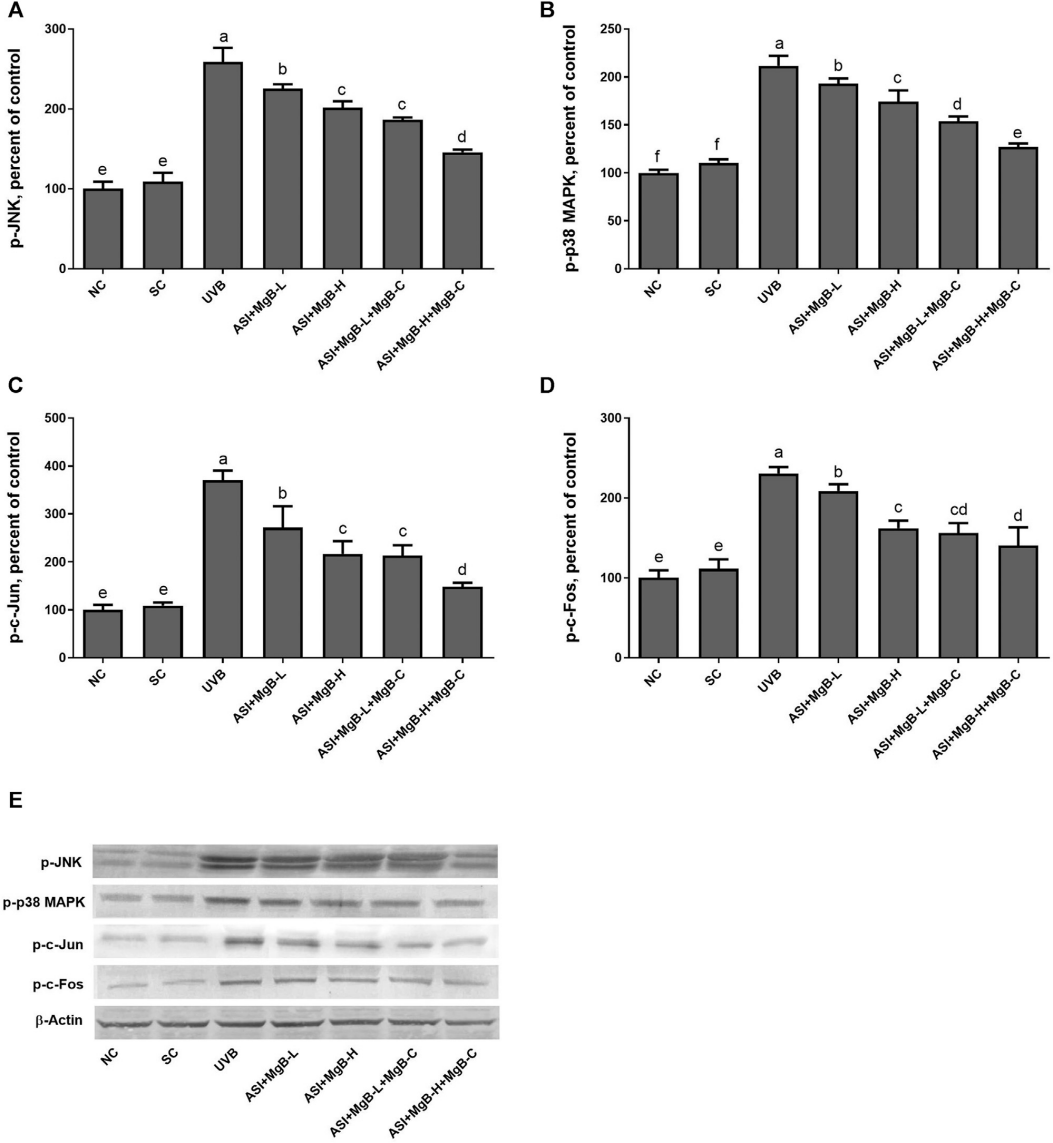
Effect of inositol-stabilized arginine silicate complex (ASI) and magnesium biotinate (MgB) on c-Jun N-terminal kinase (JNK; **A**), p38 mitogen-activated protein kinase (MAPK); **B**), AP1, activator protein 1 (c-jun; **C** and c-fos; **D**) protein levels in rats with UVB-induced photoaging. Data are expressed as a percent of the control value. Each bar represents the mean and standard error of the mean. Blots were repeated at least 3 times. Western blot analysis was performed with actin included ensuring equal protein loading **(E)**. The data are percentages of the control. **a–e**: Values within the bars with different superscripts are significantly different (One-way ANOVA and Tukey’s post-hoc test, p < 0.05).

In order to determine the mechanism of action of the combination of ASI and MgB, we examined the mitogen-activated protein kinases (MAPKs) and AP-1 signaling pathways known to be associated with MMP expression. MAPK signaling factors stimulate activator protein-1 (AP-1) expression, which is a transcription factor and consists of c-Jun and c-Fos subunits. For this purpose, we measured two major components of phosphorylated c-jun N-terminal kinase (p-JNK), p-p38, and AP-1 levels by western blot analysis. As seen in Figure 6, levels of p-JNK and p-p38 were increased by UVB exposure compared to control and shaved groups (*p* < 0.0001). ASI + MgB treatments downregulated p-JNK and p-p38 levels in rats exposed to UVB. Their levels are particularly reduced by ASI + MgB combined with MgB cream than other groups (*p* < 0.0001). Similarly, UVB irradiation significantly activated the skin tissue AP-1 (p-c-Jun and p-c-Fos) level in rats (*p* < 0.0001). However, ASI + MgB treatments, particularly with MgB cream application, blocked the activation of the AP-1 in UVB-irradiated rats (Figure 6, *p* < 0.0001).

**FIGURE 7:**
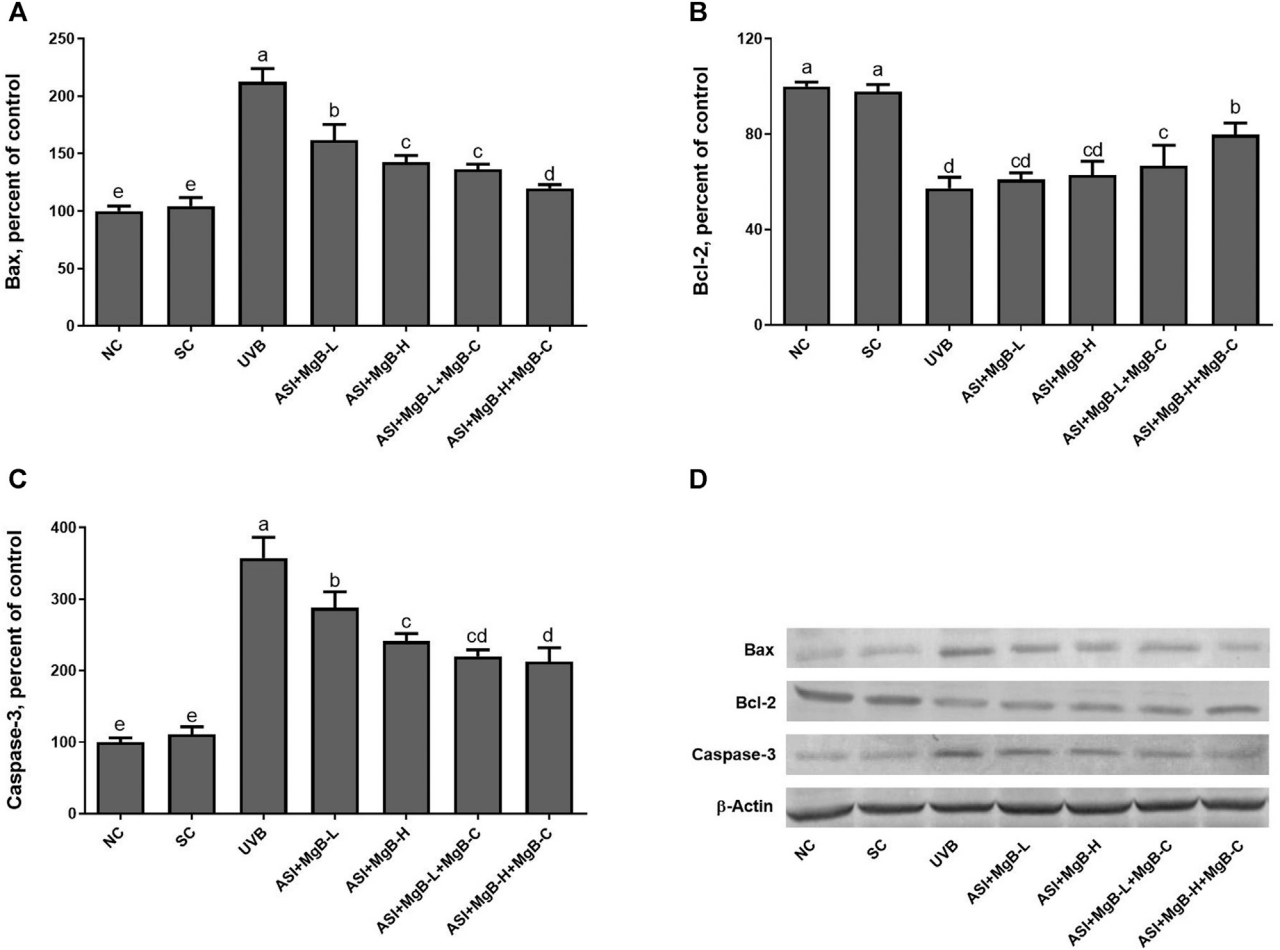
Effect of inositol-stabilized arginine silicate complex (ASI) and magnesium biotinate (MgB) on the Bax **(A)**, Bcl-2 **(B)** and caspase-3 **(C)** levels in rats with UVB-induced photoaging. Data are expressed as a percent of the control value. Each bar represents the mean and standard error of the mean. Blots were repeated at least 3 times. Western blot analysis was performed with actin included ensuring equal protein loading **(D)**. The data are percentages of the control. **a–e**: Values within the bars with different superscripts are significantly different (One-way ANOVA and Tukey’s post-hoc test, *p* < 0.05).

As shown in Figure 7, UVB exposure significantly increased the expressions of Bax and caspase-3 and decreased the Bcl-2 levels compared to the control and shaved groups (*p* < 0.001). However, ASI + MgB treatments, especially in the MgB high dose with MgB cream application, markedly reversed their levels (*p* < 0.01).

## Discussion

Photoaging is a skin condition resulting from the chronic and cumulative effects of long-term exposure to UV radiation and is characterized by epidermal thickening, accumulation of abnormal elastin‐containing material, deep wrinkles, and abnormal pigmentation (Gragnani et al., 2014). Arginine and biotin exert beneficial effects on vascular function, collagen deposition and synthesis, protein synthesis, and cell proliferation, all of which are crucial for skin health and prevent and control the photoaging process (Dorner et al., 2009; Zempleni et al., 2009; Kalman et al., 2015; Durmus et al., 2017; Mock, 2017). Thus, in the current study, we studied the protective effects of ASI and MgB (a newly produced and highly absorbed biotin salt) orally, with and without MgB topically, on preventing UVB-induced skin photoaging in rats. At the macroscopic level, we found that the skin of rats exposed to UV had a wrinkled and sagging appearance and reduced skin elasticity. At the histopathological level, degraded collagen and elastic fibers were observed. Also, increases in skin thickness, microfolds, and inflammatory cells were shown. However, oral ASI + MgB treatment, with and without MgB topical application, alleviated these skin damages by regulating biotin-dependent carboxylases, mTOR pathways, matrix metalloproteinase, and inflammatory factors. In addition, ASI + MgB treatment increased serum arginine, biotin, Mg, Fe, Zn, Cu, and Si levels, particularly the high doses of ASI and MgB combined with topical MgB cream. No previous studies have examined the protective effects of ASI and MgB treatment against UVB-induced skin damage in rats. However, *in vitro* and *in vivo* studies on the individual use of arginine, Si, biotin, and Mg (Barbul et al., 1990; Jung et al., 2015; Lipner, 2018). In a previous study, we conducted to reveal ASI's properties on wound healing, and we found that granulation tissue grew faster in ASI-treated groups than in the control group (Durmus et al., 2017). Besides, Proctor et al. (Proctor et al., 2007) demonstrated that ASI is easily and directly absorbed *in vivo*, as shown by increased plasma arginine concentrations in ASI-treated rats. Moreover, arginine supplementation has been shown to improve collagen deposition and immune function in healthy volunteers (Barbul et al., 1990). Zhang et al. (Zhang et al., 2008) reported that arginine treatment has an anabolic effect on proteins in the skin and muscle protection in the catabolic mechanism. The same authors have suggested that the mechanism of arginine-induced muscle protein anabolism may be associated with the stimulation of amino acid transport from arterial blood to the tissue-free amino acid pool, thus increasing amino acid availability for protein synthesis.

Biotinidase is an essential enzyme for biotin synthesis (Zempleni et al., 2009; Tong, 2013; León‐Del‐Río, 2019). Hydroxyproline plays a significant role in collagen stability and permits the sharp twisting of the collagen helix (Pyun et al., 2012; Kumar Srivastava et al., 2016). Earlier studies indicate that exposure to UV radiation reduces elastin formation and increases elastase activity, thereby leading to elastin degradation (Mora Huertas et al., 2016; Weihermann et al., 2017). Consistent with previous reports, we showed that UVB exposure induced a reduction in the concentration of hydroxyproline and activity of biotinidase and increased elastase activity, an enzyme that breaks down elastin, in the dorsal skin. However, as a result of treatment with ASI + MgB, hydroxyproline concentration and biotinidase activity improved, while elastase activity was significantly reduced. In support of these results, the morphology and density of collagen and elastic fibers were similar. Similarly, in a previous study, the hydroxyproline and collagen content detected in the area of a wound increased in ASI-treated rats (Durmus et al., 2017). In the current study, the ASI + MgB-H + MgB-C treatment was the most effective treatment concerning eliminating the adverse effects of UVB radiation.

The excessive reactive oxygen production (ROS) during UV radiation-induced skin damage causes an imbalance between prooxidant production and antioxidant defense, lipid peroxidation, DNA damage, activation and expression of proteins, and infiltration of the inflammatory cells (Darr and Fridovich, 1994). MDA is an important end-product of lipid peroxidation and acts as an indicator of the presence of ROS (Zhang et al., 2013). Therefore, the MDA levels were measured in the dorsal skin samples. In the present study, treatment with ASI + MgB and MgB cream has been shown to reduce MDA formation in the skin tissue of rats. This indicates that the antioxidant mechanism of MgB and ASI may involve the upregulation of endogenous antioxidants, thus preventing lipid peroxidation. Although there are no studies on the effects of the combination of ASI and MgB on MDA levels in skin rats, several studies suggest that Mg improves antioxidant status, confirming these results (Mills et al., 1986; Yavuz et al., 2013).

In the current study, UVB irradiation decreased the skin activities of the biotin-dependent carboxylases (ACC1, ACC2, PC, PCC, and MCC). These carboxylases participate in different metabolic pathways, including fatty acid synthesis, metabolism of amino acids, cholesterol, and odd-chain fatty acids, gluconeogenesis, and tricarboxylic acid anaplerosis (Riveron-Negrete and Fernandez-Mejia, 2017). However, ASI + MgB administration prevented the decrease of skin ACC1, ACC2, PC, PC, PCC, and MCC levels in rats exposed to UVB. To our knowledge, the current findings are among the first to show the practical effects of ASI + MgB in stimulating biotin-dependent carboxylases in the skin of UVB-exposed rats. Rathman et al. (Rathman et al., 2003) reported that carbamazepine reduced specific activities and abundance of biotinylated PC and ACC in the brain and liver, whereas biotin protected these reductions in ACC. In another study, biotin supplementation to biotin-deficient primary culture hepatocytes or biotin-deficient chicks increased PC activity (Rodríguez-Meléndez et al., 1999).

The mTOR signaling pathway is essential for cell growth, cell proliferation, angiogenesis, protein translation, and apoptosis (Wang et al., 2016; Chiu et al., 2017). Exposure to UVB is a significant environmental risk factor for skin damage, characterized by abnormal activation of Akt/mTOR (Carr et al., 2012). Similarly, numerous studies have shown that the mTOR pathway involved in protein synthesis and cell division is upregulated by UVB in the skin (Huang et al., 2002; Carr et al., 2012). In the present study, ASI + MgB treatment suppressed the UVB-induced mTOR pathway in the dorsal skin of rats. This is the first study to demonstrate the effects of ASI + MgB on mTOR/p70S6K signaling in UVB irradiation skin. However, arginine, an active compound in the ASI complex, has been reported to modify the p70S6K activity and p-4E-BP1 through the mTOR pathway (Ban et al., 2004). In addition, Elahi et al. (Elahi et al., 2018) reported that the expression of p-mTOR was significantly upregulated in biotin-deficient CD4 ^+^ T cells compared with the sufficient group and was inhibited by rapamycin. They also noted that p70S6 kinase exhibited significantly upregulated expression in biotin-deficient CD4 ^+^ T cells compared with the adequate group (Elahi et al., 2018). In a prior study, Jiang et al. (Jiang et al., 2017). reported that autophagy was induced by cyclophosphamide challenge as demonstrated by increased LC3, p70S6K, and 4EBP1 levels, while magnesium isoglycyrrhizinate well reversed these changes *in vivo* and *in vitro*.

Many studies have reported that matrix metalloproteinases (MMPs) are the primary enzyme responsible for protein and collagen degradation in the skin and are stimulated by UVB irradiation (Lee et al., 2009; Quan et al., 2009). In the present study, MMP-1 and MMP-3 levels in rats that received ASI + MgB, particularly high dose ASI + MgB with MgB cream, were lower than rats exposed to UVB irradiation. This decrease in MMP-1 and MMP-3 can be attributed to the inhibition of collagen degradation enzymes in animals receiving ASI + MgB. Although there are no other studies that examined the effects of ASI and MgB in combination on MMPs, in an earlier study, it was shown that MMP-2 and MMP-9 expressions decreased in skin wound areas in ASI-treated groups (Durmus et al., 2017). Also, Yue et al. (Yue et al., 2004) reported that magnesium reduced MMP-2 production dose-dependently.

Previous studies have reported that UVB induces NF-κB activation through the skin’s cell surface receptors, resulting in overexpression of proinflammatory cytokines including TNF-α, IL-1, IL-6, NFκB, and AP-1 (Abeyama et al., 2000; Aubin, 2003; Syed et al., 2012; Kwon et al., 2013; Kong et al., 2015). In the present study, ASI + MgB treatment inhibited the overproduction of TNF-α, NFκB, IL-6, IL-8, and COX-2 caused by UVB, as well as suppressed the UVB-induced dermal inflammatory infiltrates. This effect was particularly strong with the use of MgB in high doses and MgB cream combined with ASI. In previous studies, we demonstrated the anti-inflammatory effects of the ASI complex (Durmus et al., 2017; Sahin et al., 2019). For instance, the ASI complex inhibits the release of proinflammatory and anti-inflammatory cytokines such as IL-6, TNF-α, and IL-1 at the wound site, contributing to effective wound closure (Durmus et al., 2017). In addition, Elahi et al. (Elahi et al., 2018) reported that IFN-γ and IL-17 production was significantly higher in biotin-deficient CD4 + T cells than in the adequate group. Furthermore, a human study indicated that high doses of biotin intake inhibit the proliferation of cytokines (IL-1 and IL-2) and peripheral blood mononuclear cells (Zempleni et al., 2001). Moreover, magnesium isoglycyrrhizinate suppressed the levels of TNF-α, IL-1β, IL-6, antioxidant enzymes (SOD, GSHPx), iNOS, and caspase-3 in ischemia-reperfusion injury model rats (Zhao et al., 2017). Magnesium chenoursodeoxycholic acid (Mg-CUD) suppression increases MMP-2 and tissue inhibitor of MMP-1 mRNA expression, as well as levels of NF-κB, TNF-α, IL-6, and COX-2 in carbon tetrachloride-induced liver fibrosis in rats (Kang et al., 2012).

DNA damage or ROS caused by UVB often triggers certain signaling pathways, such as MAPKs, which in turn regulate a nuclear transcription factor AP-1, known to play a role in the proliferation and survival of cells (Torres and Forman, 2003). Phosphorylated MAPKs lead to increased expression of c-Jun and c-Fos and then activate AP-1 (Angel et al., 2001). Activated AP-1 stimulates MMP expression and degrades collagen (Lu et al., 2016), (Quan et al., 2009). Recent studies have shown that activation of MAPKs such as p-JNK and p-p38 MAPK is tightly correlated with inflammation and the development of skin damage through increased expression of COX-2 (Mahns et al., 2004). Moreover, it is well reported that AP-1 plays a key role as a transcription factor involved in UVB-induced COX-2 expression in many systems (Abbas et al., 2016). In the present study, ASI + MgB treatment significantly suppressed p-JNK, p-38, c-Jun, and c-Fos in UVB-irradiated skin tissues. These results suggest that ASI + MgB treatment suppresses the levels of MMPs, and proinflammatory cytokines by regulating the AP-1 and MAPK pathways.

UV radiation induces apoptosis through imbalanced Bcl-2 proteins family and activated caspases (Cryns and Yuan, 1998). The Bcl-2 protein family plays a critical step in pro-apoptotic and anti-apoptotic effects by regulating the permeability of the mitochondrial membrane (Tait and Green, 2010). Caspases may block the cell cycle, label apoptotic cells, breakdown structural proteins in the cytoskeleton, and inactivate DNA repair enzymes, leading to apoptosis (Chandra et al., 2000). In the present study, we found that the levels of Bax and caspase-3 were significantly increased in the UVB-irradiated skin tissues compared to the control and shaved groups, whereas ASI + MgB treatment, particularly the administration of ASI + MgB in high dose with ASI + MgB cream application, significantly reversed the increase of Bax and caspase-3 in UVB-irradiated skin tissues. Compared with the control group, the UVB exposure decreased Bcl-2, while the ASI + MgB treatments prevented this UVB-induced trend. The above results concluded that ASI + MgB treatments, ASI + MgB in high dose with ASI + MgB cream could modulate the levels of Bax, Bcl-2, and caspase-3 proteins in the skin of rats receiving UVB irradiation. However, there are no previous studies to compare these results regarding the effects of the combination of ASI and MgB treatments on apoptosis in rats.

The present study results indicated that the combination of ASI complex and MgB, particularly with ASI, high-dose MgB, and MgB cream, reduced UV-induced skin photoaging by anti-inflammatory processes. This combination regulates MMPs, mTOR, pathways, and inflammatory markers, including TNF-α, NFκB, IL-6, IL-8, COX-2 (may be associated with suppression of JNK, p38 MAPK, and AP-1) and apoptosis. Based on these findings, it is possible that ASI and MgB could be useful in sun-protective treatments.

## Data Availability

The original contributions presented in the study are included in the article/Supplementary Material, further inquiries can be directed to the corresponding author.
