# Supplementary material for: The Protective Effects of a Combination of an Arginine Silicate Complex and Magnesium Biotinate Against UV-Induced Skin Damage in Rats
Source: Front Pharmacol. 2021 Jun 15;12:657207. doi: 10.3389/fphar.2021.657207 (PMC8250765; doi:10.3389/fphar.2021.657207)
Supplement: Supplementary file 1 [file DataSheet1.pdf]

Supplementary Material

1. Supplementary Figures

Supplementary Figure Legends

Supplementary Figure 1| Effect of inositol-stabilized arginine silicate complex (ASI) and magnesium biotinate (MgB) on macroscopic (**A**) and visual appearances (**B**) in rats with UVB-induced skin photoaging. Each bar represents the mean and standard error of the mean. a-e: Values within the bars with different superscripts are significantly different (One-way ANOVA and Tukey's *post-hoc* test, $P < 0.05$).

Supplementary Figure 2| Effect of inositol-stabilized arginine silicate complex (ASI) and magnesium biotinate (MgB) on macroscopic changes. In macroscopic images, the groups are shown as **a:** NC, normal control; **b:** SC, shaved control; **c:** UVB; d: ASI+MgB-L; **e:** ASI+MgB-H; **f:** ASI+MgB-L+MgB-C; **g:** ASI+MgB-H+MgB-C.

Supplementary Figure 3| Effect of inositol-stabilized arginine silicate complex (ASI) and magnesium biotinate (MgB) on skin elasticity as evaluated by pinch testing in rats with UVB-induced skin photoaging. Each bar represents the mean and standard error of the mean. a-d: Values within the bars with different superscripts are significantly different (One-way ANOVA and Tukey's *post-hoc* test, $P < 0.05$).

Supplementary Figure 4| Western blot bands for Figures 3 (Panel A), 4 (Panel B), 5 (Panel C), 6 (Panel D), and 7 (Panel E). Blots were repeated at least 3 times. Two bands (number 2 and 3) belonging to each group are shown in this Figure. The other representative band for each protein level was shown in Figures 3, 4, 5,6, and 7.

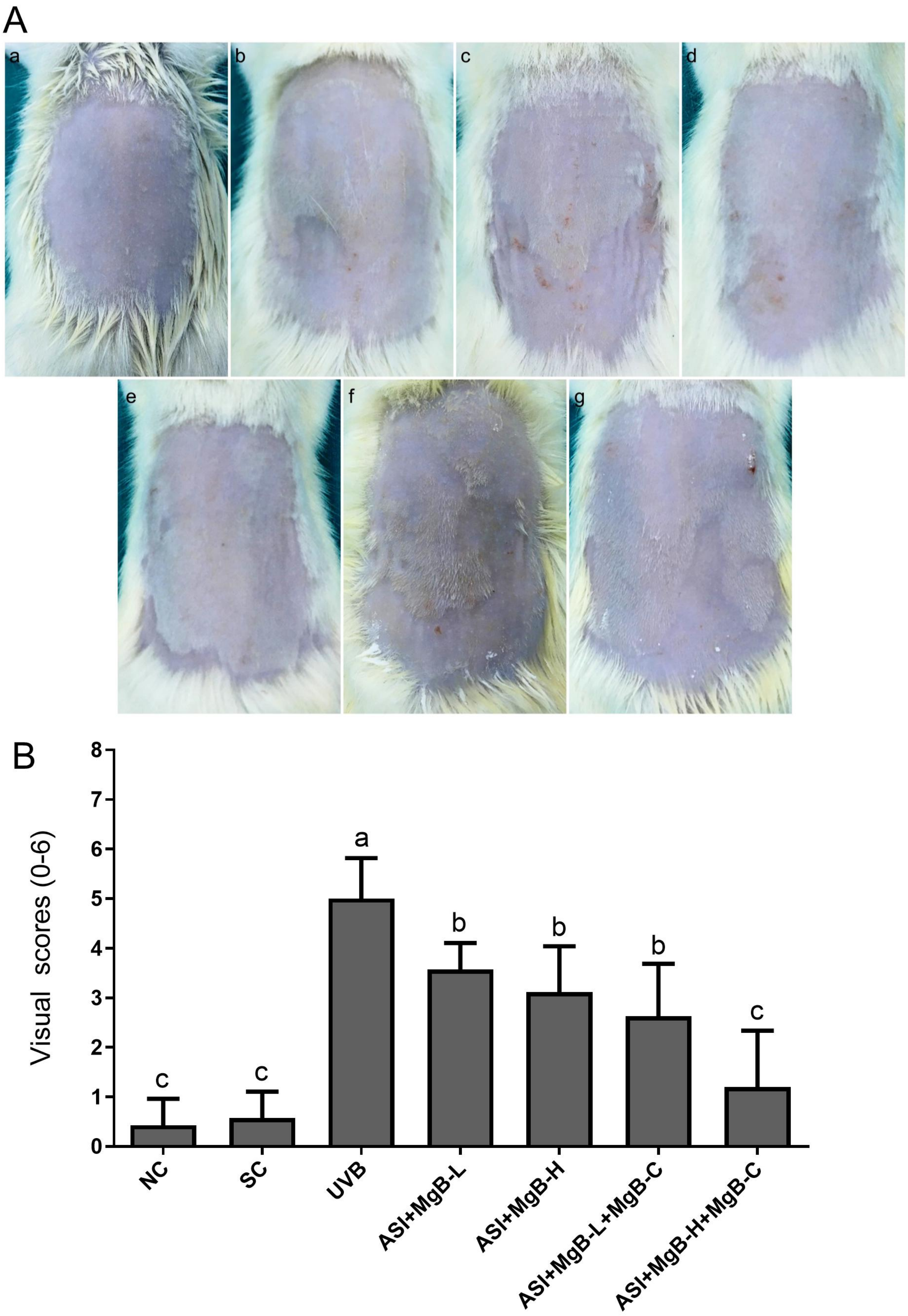

Supplementary Figure 1

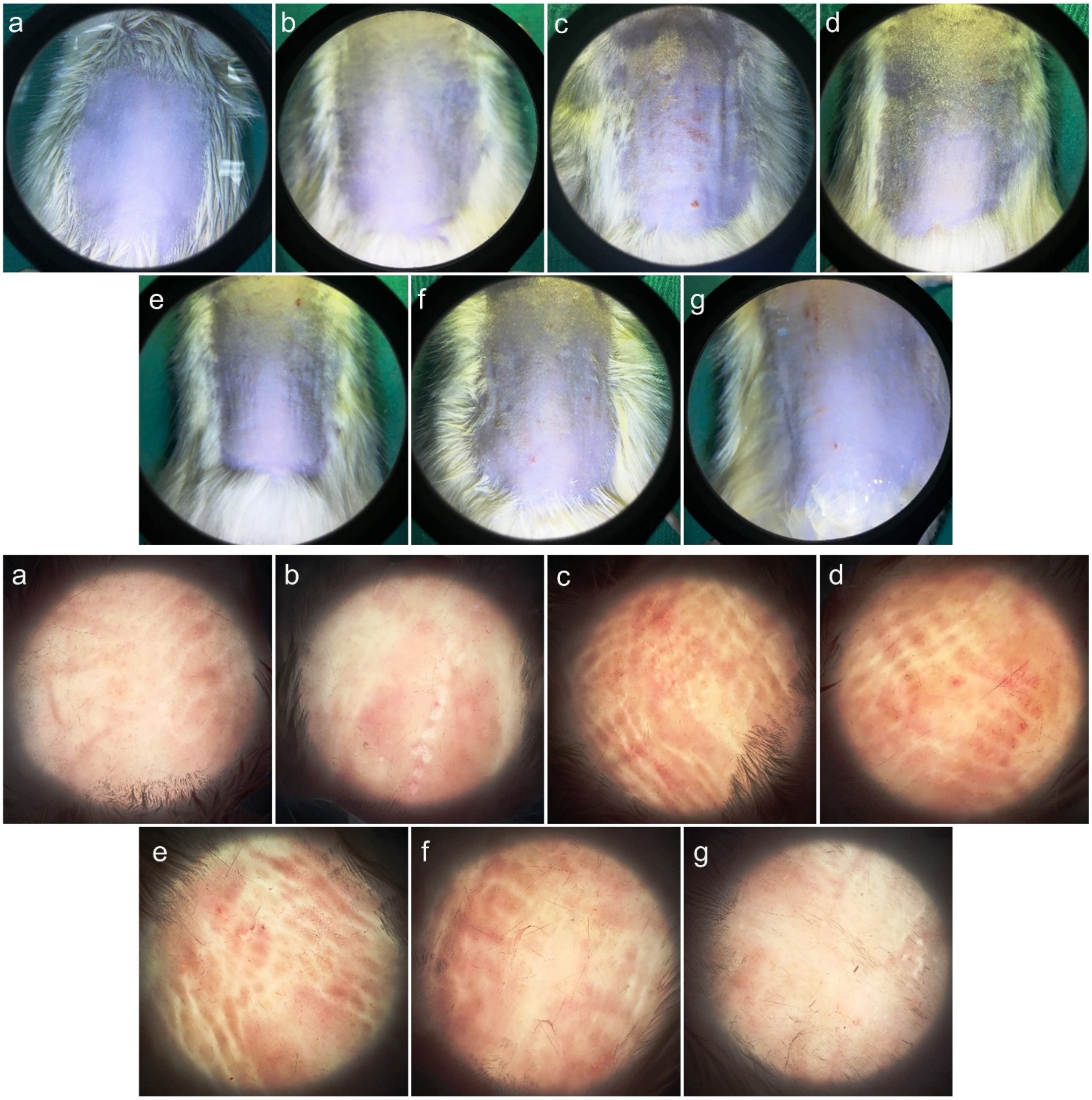

Supplementary Figure 2

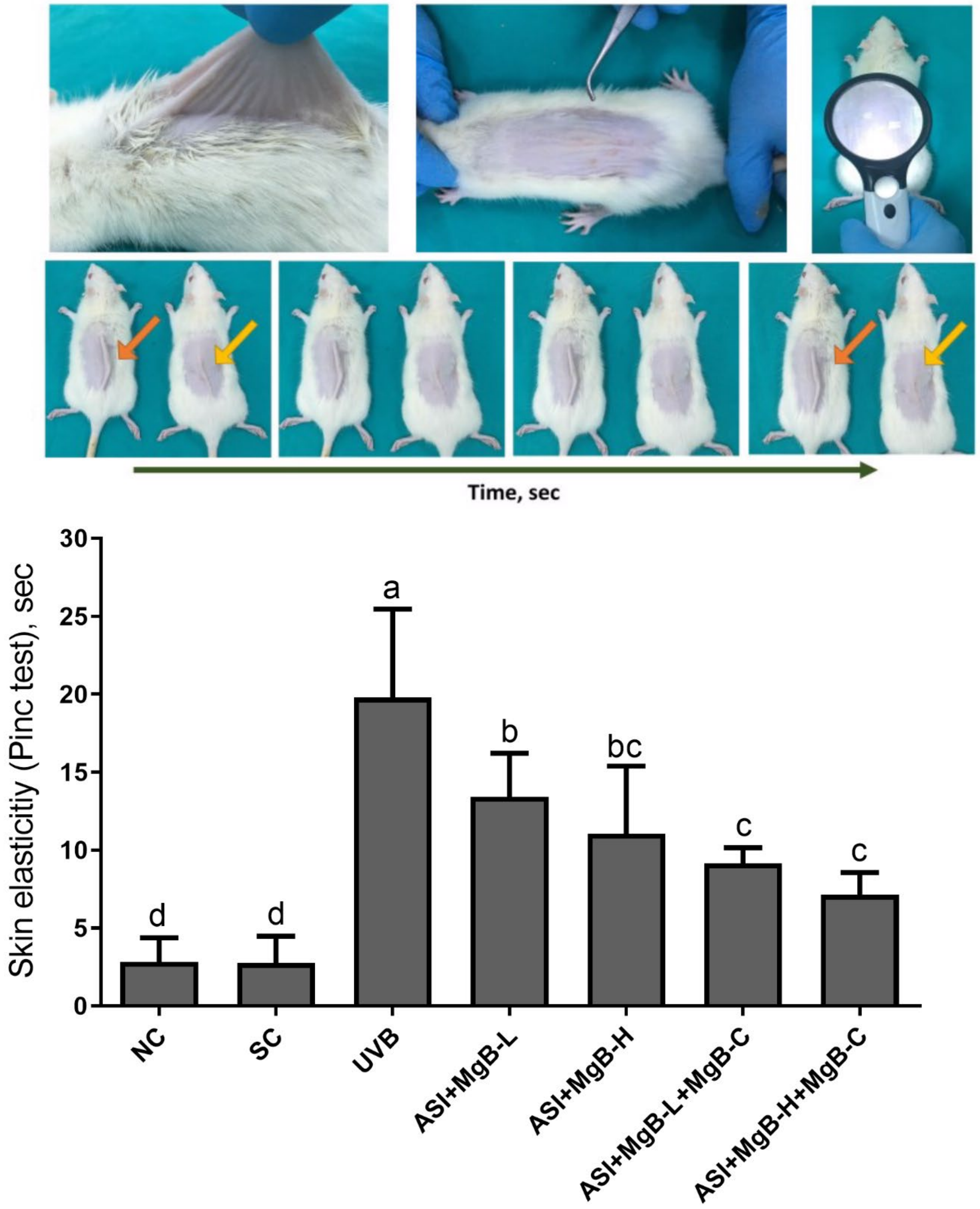

Supplementary Figure 3

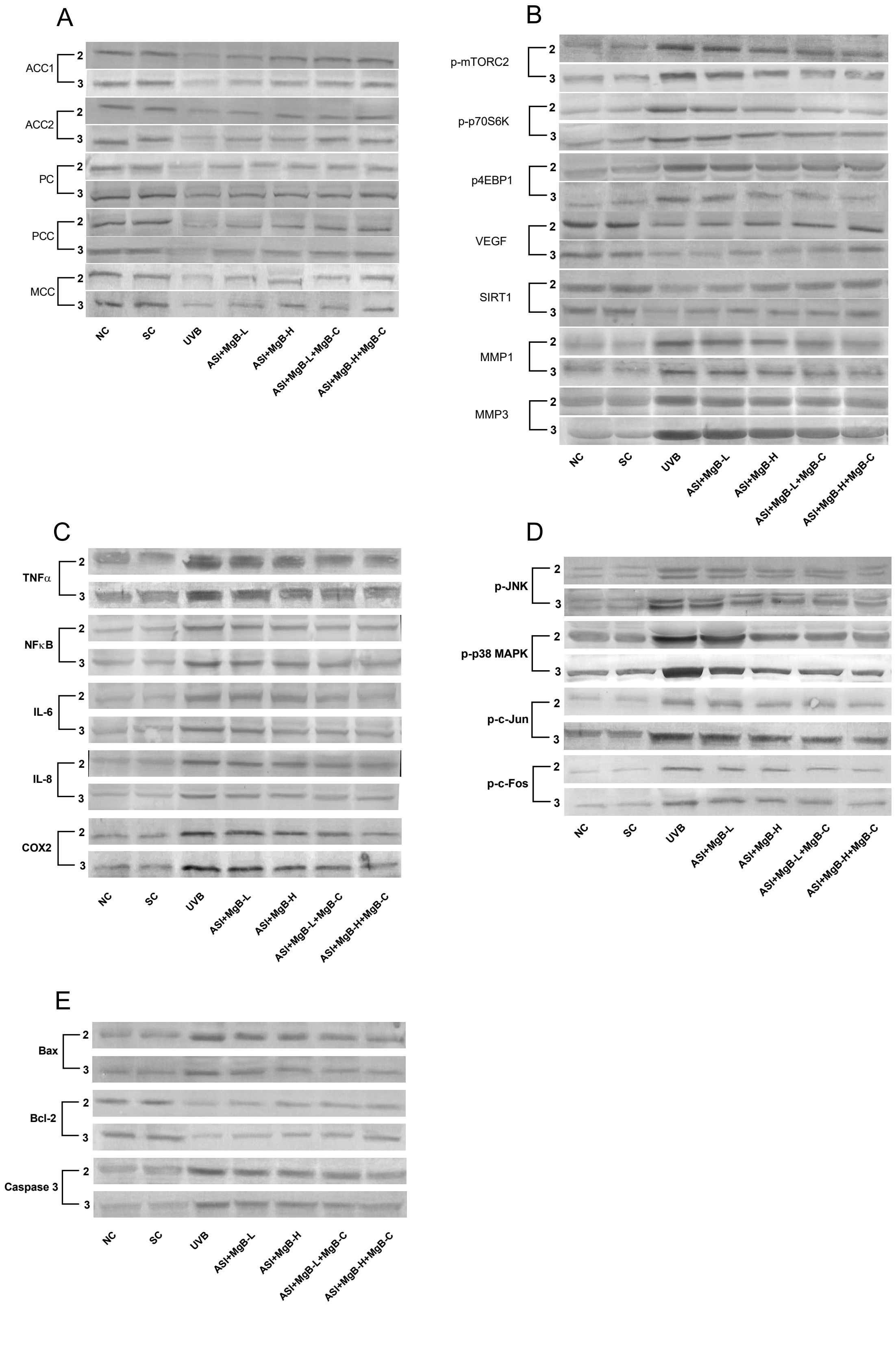

Supplementary Figure 4

Supplementary Table 1| Treatment schedule of the study**.**

Groups	Shave	UVB radiation	Arginine silicate inositol (ASI; 4.14 mg/rat/d -140 mg human equivalent	Magnesium Biotinate		
				Low (48.7 micg/rat/d-1.5 mg human equivalent	High (325 micg/rat/d -10 mg human equivalent)	Cream
Normal control (NC)	-	-	-	-	-	-
Shaved control (SC)	+	-	-	-	-	-
Shaved+UVB (UVB)	+	+	-	-	-	-
Shaved+UVB+ASI+MgB low dose (ASI+MgB-L)	+	+	+	+	-	-
Shaved+UVB+ASI+MgB high dose (ASI+MgB-H)	+	+	+	-	+	-
Shaved+UVB+ASI+MgB low dose+MgB Cream (ASI+MgB-L+MgB-C)	+	+	+	+	-	+
Shaved+UVB+ASI+MgB high dose+MgB cream (ASI+MgB-H+MgB-C)	+	+	+	-	+	+

Supplementary Table 2| The grading scale for evaluation of photoaging [46]

Grade	Evaluation criteria
**0**	No wrinkles or laxity; fine striations running the length of the body
**1**	Fine striations
**2**	The disappearance of all fine striations
**3**	Shallow wrinkles
**4**	A few deep wrinkles and laxity
**5**	Increased deep wrinkles
**6**	Severe wrinkles; development of tumors/lesions

0: normal skin, 6: severely photo-damaged skin.
